# Research on the Effects of Occupational Stress and the *DRD2* Gene on the Psychological Health of Workers in the Xinjiang Desert Oil Field

**DOI:** 10.3389/fpsyt.2021.737228

**Published:** 2021-09-14

**Authors:** Ting Jiang, Gulijianati Wumaier, Xue Li, Xu Yang, Jiwen Liu

**Affiliations:** Department of Public Health, Xinjiang Medical University, Urumqi, China

**Keywords:** mental health, *DRD2* gene, occupational stress, health, mental

## Abstract

**Background:** This study investigated the relationship between occupational stress and the mental health of people working in oil fields in the arid desert environment of Xinjiang, and revealed the causal relationship between occupational stress and psychological disorders, while furthermore exploring the relationship between psychological disorders and genetic levels.

**Methods:** The participants of this study included oil field company workers from the Xinjiang Petroleum Administration of Karamay City, Xinjiang, who underwent occupational health examinations. The Occupational Stress Inventory Revised Edition (OSI-R) was used to measure the occupational stress of the oil workers. The mental health status of oil workers was evaluated using the Symptoms Checklist-90.

**Results:** Occupational tasks: The total scores of the personal strain and mental health questionnaires were positively correlated with somatization, obsessive-compulsive symptoms, interpersonal sensitivity, depression, anxiety, hostility, terror, paranoia, and psychosis *(P* < 0.05). Individual coping resources and the mental health total score was negatively correlated with somatization, obsessive-compulsive symptoms, interpersonal sensitivity, anxiety, hostility, terror, paranoia, and psychosis. The following factors were identified as mental health risk factors: female gender; age 45 and above (relative to ≤30 years old); high scores on the personal strain questionnaire; occupational stress; external effort; internal investment; and high effort-low return. The following factors were identified as protective factors for mental health: Han nationality; oil transportation (relative to drilling); individual resilience; and work returns. In respect to the abnormal psychological group and the normal psychological group, statistically significant differences were found in the distribution of genotypes and allele frequencies at the rs1800497 locus (*P* < 0.05). The depression and paranoia scores observed between different genotype groups at the rs1800497 locus were statistically significant (*P* < 0.05).

**Conclusions:** This study shows that occupational stress and the *D2* dopamine receptor (*DRD2*) gene have an impact on the mental health of oil field workers in the arid desert environment of Xinjiang. Effort-reward imbalance and occupational stress were identified as risk factors for mental health, while rewards for work were protective factors. Higher levels of occupational stress may lead to depression and other psychological disorders, adversely affecting mental health. In oil field operators in the arid desert environment of Xinjiang, the AA genotype of the *DRD2* gene in the rs1800497 locus was identified as a genotype specific to susceptibility to mental health problems, and a correlation was found between the A allele and an increased risk of psychological problems. Therefore, it is necessary to devise relevant measures to alleviate occupational stress among oil workers and increase their job rewards, so as to improve their mental health.

## Introduction

Mental health problems are becoming increasingly serious worldwide. According to the report titled *Depression and Other Common Mental Disorders* released by the World Health Organization (WHO) in 2017, the average international incidence rate of depression is about 4.4% (1). In China, the White Paper on the *Mental Health of Chinese Urban Residents*, which was released in 2018 and surveyed more than 1.13 million people, found that 73.6% were in a state of sub-mental health, while only 10.3% of urban residents were in a state of good mental health (2). In today's society, with rapid economic development and increasingly fierce competition, people often experience high levels of work-related pressures and work overload. Obviously, occupational pressure and tension also greatly affect mental health. As the traditional medical model has gradually shifted to the biological-psychosocial model (3), more and more scholars, both at home and abroad, are focused on examining the relationship between occupational stress and mental health. Previous studies have confirmed that medical workers, oil workers, and other special occupational groups generally exhibit a high level of occupational stress (4–7), which affects their mental health to different degrees (8, 9). Oil field workers belong to a special working group, their tasks are often repetitive, and they work in the Gobi Desert, which creates little opportunity for recreational activities and family reunions. These factors often result in higher levels of occupational stress, and studies have found that long-term occupational stress adversely affects mental health (10, 11). In a study that investigated the relationship between occupational stress and the mental health of oil workers, it was found that higher levels of occupational stress led to poorer mental health (12).

The causes of mental illness are often complex. In addition to simple environmental factors, such as excessive work pressure, job security, role conflict, job demands, job control, social supports, and so on (13–16), the impact of genetic factors on mental health, and the interaction between genes and the environment have also become a focus of researchers (17, 18). From the perspective of a gene-environment interaction, if an individual in a specific candidate environment carries some sensitive genes, environmental factors will greatly increase the impact on the individual's mental health. Jiang et al. analyzed the DNA methylation level of the monoamine oxidase A (*MAOA*) gene in their study on the mental health of oil workers in arid deserts in Xinjiang, and the findings indicated that the methylation state may affect mental health (19). A number of studies have also pointed out that the *MAOA* gene is associated with depression, anxiety, and stress-related disorders, as well as obsessive-compulsive disorder and other psychological disorders (20–22). In a cross-sectional study of a large number of Chinese adolescents with depression, it was found that catechol-O-methyltransferase (*COMT*) gene VAL158MET polymorphism, *DAT1* gene rs27072 polymorphism, and the peer relationship played an important role in adolescent depression symptoms (23). In studies of healthy adults, Klaus et al. found that early life stress, combined with the interaction of environmental and genetic factors, influenced individual cognitive functioning, and the *COMT* gene and the *D2* dopamine receptor (*DRD2*) gene mutation may increase the risk of mental illness, such as schizophrenia (24, 25). In addition to playing an important role in the pathogenesis of psychological diseases, such as anxiety and depression, the *DRD2* gene also plays a certain role in the pathogenesis of chronic diseases, such as sleep disorders (26, 27).

In conclusion, genetic factors play an important role in the occurrence and development of mental health problems under specific circumstances. At present, there are many research studies on the relationship between occupational stress and psychological disorders, but simple epidemiological investigations or simple genetic analyses are not enough to explain the relationship between occupational stress and psychological disorders. However, in terms of research on the effect of occupational stress on mental health, few studies have been carried out to examine the role of genetics, which is worthy of further study, as such research can elucidate an understanding of the etiology of mental health problems caused by occupational stress. Therefore, this study investigated the relationship between occupational stress and the mental health status of oil field workers working in arid desert environment of Xinjiang, and revealed the causal relationship between occupational stress and mental disorders, so as to further explore the relationship between psychological disorders and genetics.

## Methods

### Participants

This study was carried out at the Center for Disease Control and Prevention and the Central Hospital of Karamay City. The questionnaire was conducted in conjunction with the physical examination of the oil workers. The survey period was from March 2017 to June 2018. The participants in this study included employees of the Xinjiang Petroleum Administration Bureau of Karamay City, Xinjiang, China, and the National Petroleum Corporation (CNPC). The administration consisted of 25 sub-units and ~150,000 employees who were engaged in all types of jobs related to the oil industry. Based on the standard industry classification of the CNPC, four operating areas, four production plants, and six E&P companies were selected using a three-stage stratified sampling method. Then, based on company size, a large company (>400 workers) and a small company (<400 workers) were randomly chosen from the selected business area; 600 employees from the large company and 300 employees from the small company were randomly selected. A large company (>1,000 workers) and a small company (<1,000 workers) were selected from the production plant. For the production plant, 1,500 employees from a large company and 800 employees from a small company were randomly selected. One large company (>200 workers) and two small companies (<200 workers) were selected from the E&P company; 300 people were randomly selected from the large company and 150 people were selected from each small company. After communication and consultation with the hospital, relevant information was obtained from the medical examiner of each company before the health examination. Furthermore, the number of participants who underwent medical examinations (from 1… Start) was recorded and this number was input into SPSS for Windows version 22.0 (SPSS Inc, Chicago, IL, USA). The inclusion criteria included the following: oil workers (aged between 18 and 60 years old, who had been employed for more than 1 year). A total of 3,800 questionnaires were retrieved, of which 3,631 were deemed valid, with an effective recovery rate of 95%. In respect to gene measurement, case-control studies were used to determine the sample content, and the participants were evaluated according to the health checklist. Thereafter, 20% of the participants who had completed the questionnaire were randomly selected as the experimental research objects. Therefore, 726 participants were randomly selected as subjects for the molecular biology portion of the trial. In instances in which blood samples were not collected, such samples, as well as those that did not meet the DNA extraction criteria, were excluded. Finally, the *DRD2* genotype and allele distribution of 696 samples were tested. The study protocol was approved by the Ethics Committee of Xinjiang Medical University, and all participants voluntarily provided their written informed consent prior to the investigation.

### Measurement of Occupational Stress

The Chinese Revised Edition of the Occupational Stress Inventory (OSI-R), which is more suitable for the Chinese population, was adopted in this study, and the scale has high reliability and validity among all occupational groups in China (28–30). The scale included three subscales: Occupational Role Questionnaire (ORQ), Personal Strain Questionnaire (PSQ), and Personal Resources Questionnaire (PRQ). Responses were measured using a scale ranging from 1 to 5. The occupational task subscale consisted of six dimensions (i.e., task overload, task discomfort, task ambiguity, task boundary, responsibility, and work environment), which were used to evaluate individual levels of occupational stress. Higher scores indicated higher stress levels. The subscale of individual stress responses consisted of four dimensions (i.e., the business stress response, psychological stress response, interpersonal stress response, and body stress response), which were used to assess the level of individual stress responses. Higher scores indicated higher stress levels. The subscale of individual resilience was composed of four dimensions (i.e., leisure, self-care, social support, and rational coping) to evaluate the participant's coping ability. Higher scores were taken to indicate lower levels of stress and a greater ability to cope.

### Measurement of Mental Disorders

This study used the Symptoms Checklist 90 (SCL-90) to evaluate the mental health status of desert oil field workers. This scale was based on the Symptoms Checklist 90 (Hopkins Symptom Checklist, 1973) compiled by Derogatis, and mental symptoms were assessed using a self-rated scale which consisted of a total of 90 items (31). The subscale contained nine factors, which included somatization, compulsive symptoms, interpersonal sensitivity, depression, anxiety, hostility, fear, paranoia, psychosis, as well as another nine factors. Responses were measured using a scale ranging from 1 to 5, such that 1 = none, 2 = mild, 3 = moderate, 4 = severe, and 5 = severe. The total score was obtained from the sum of the scores for all of the items, and the total score of each factor item was obtained by the sum of the factors. A total score of more than 160 points or a factor score of more than 2 points was taken to indicate a psychological disorder. This scale has shown high reliability and validity among all groups (32, 33). In respect to the measurement results, the present study referred to Jinhua's et al. research, which obtained the data of 1,388 normal people from 13 regions in China, and this was taken as the norm (34).

### *DRD2* Gene Determination

Prior to the physical examination, the participants were required to abstain from a high-fat diet and alcohol for 3 days. After fasting, blood samples (5 ml) were taken as part of the physical examination. That day, following the collection of all blood samples, the samples were centrifuged at 3,500 rpm for 7 min to separate serum and plasma, and they were stored at −80°C. Genomic DNA was purified from the samples using a whole blood genome extraction kit and cryopreserved at −20°C until use. Based on the relevant literature and the Chinese population genome SNPS genotype information (CHB) obtained from the international HapMap database, we selected the rs1799732 and rs1800497 loci of the *DRD2* gene using Haploview software (http://www.broad.mit.edu/mpg), in accordance with the current internationally recognized gold standard: *R*^2^ = 0.8, MAF > 15%. The primers listed in Table 1 were genotyped by performing Snapshot SNP analysis. The data were analyzed using GeneMapper Software version 4.1 (Applied Biosystems, Foster City, CA, USA). For quality control, 5% of the randomly selected samples were subjected to secondary genotyping.

**Table 1:** PCR primer sequences.

**Primer**	**Direction**	**Sequence**
*DRD2*rs1799732	F	5′-CCCCACCAAAGGAGCTGTACCT-3′
	R	5′-ATGCGGACCTCTTCCAACACCT-3′
*DRD2*rs1800497	F	5′-GGCAACACAGCCATCCTCAAAG-3′
	R	5′-TCTCGGCTCCTGGCTTAGAACC-3′

### Quality Control

All questionnaires were distributed to the oil workers who were identified as the study subjects, and collected on-site. The questionnaire was completed anonymously within 15 min. At the initial stage of the survey, the trained investigators instructed the participants about how to complete the questionnaire, so that they could fully understand the significance of the research and ensure their active cooperation, which also encouraged the participants to truthfully and accurately complete each item of the questionnaire. During the investigation, two investigators conducted a comprehensive review of the completed questionnaires to ensure that any errors in the questionnaires were corrected promptly, and that the missing items were added and completed in time, which also safeguarded the authenticity and effectiveness of the respondents' answers. During the data entry stage, this questionnaire was independently recorded and checked by two people, and relevant logical checks were carried out. After the data were inputted, statistical software was used to randomly check and recheck the database (i.e., according to the proportion of 20%) to ensure its accuracy.

### Statistical Analysis

The two researchers independently recorded the results in a database and tested their consistency. Data were analyzed using SPSS for Windows version 22.0. Normal measurement data used means ± standard deviation statistical descriptions. Non-normal measurement data, which were expressed using median values (quartile), indicated the level of normality. In the two groups, variances in the data were identified by comparing the means and carrying out two independent samples *t*-tests. We carried out multiple comparisons of means and a single factor analysis of variance (ANOVA). If, overall, there were differences, the least significant difference (LSD) test was performed, which involved a *t*-test to compare the two groups. In the event that there was non-conformity with normality, non-parametric variance-based methods were used for the statistical analysis. The frequency and composition ratio were used for statistical description purposes, and the chi-square test was used for the statistical analysis. The non-parametric test was used for comparisons of ordered data. A partial correlation analysis was carried out to analyze the correlation between the variables, and multivariate analysis was performed using unconditional logistic regression. The significance level was set at 0.05 (bilateral).

## Results

### Comparison of Mental Health Status in Different Stress Intensity Groups

Statistically significant differences were observed in the total mental health score and the scores of all of the factors in different stress intensity groups (*P* < 0.05), which suggested differences in the following scores in different stress intensity groups: the total mental health score, somatization, obsessive symptoms, interpersonal sensitivity, depression, anxiety, hostility, terror, paranoia, and psychosis. According to the average rank, the total score and the scores of various factors related to mental health in the high stress group were the highest, followed by the moderate stress group and the low stress group (Table 2).

**Table 2:** Comparison of the mental health of different stress groups.

**Variables**	**Low** **occupational stress**	**Moderate** **occupational stress**	**High** **occupational stress**	***χ*** ^2^	***P***
SCL-90 total score	113.96 ± 32.63	126.70 ± 37.33	140.36 ± 7.46	125.417	<0.001
Somatization	1.31 ± 0.47	1.45 ± 0.47	1.61 ± 0.59	95.415	<0.001
Forced symptoms	1.23 ± 0.37	1.35 ± 0.46	1.49 ± 0.56	93.917	<0.001
Interpersonal sensitivity	1.29 ± 0.43	1.44 ± 0.48	1.59 ± 0.58	91.111	<0.001
Depression	1.30 ± 0.38	1.50 ± 0.47	1.67 ± 0.57	132.742	<0.001
Anxiety	1.27 ± 0.39	1.41 ± 0.45	1.57 ± 0.55	127.06	<0.001
Hostile	1.22 ± 0.33	1.31 ± 0.44	1.44 ± 0.54	66.962	<0.001
Phobia	1.22 ± 0.37	1.35 ± 0.47	1.51 ± 0.58	99.269	<0.001
Paranoid	1.11 ± 0.34	1.25 ± 0.41	1.38 ± 0.48	104.021	<0.001
Psychotic symptoms	1.26 ± 0.40	1.45 ± 0.46	1.55 ± 0.55	69.209	<0.001

### Partial Correlation Analysis Between Occupational Stress and Mental Health Status

After controlling for confounding factors, such as gender, ethnicity, age, length of service, type of work, educational level, professional title, shift status, monthly income, marital status, and smoking status, etc., occupational tasks and individual stress responses were positively correlated with the total mental health score, somatization, obsessive symptoms, interpersonal sensitivity, depression, anxiety, hostility, terror, paranoia, and psychosis (*P* < 0.05). Individual coping resources were negatively correlated with the total mental health score, somatization, obsessive-compulsive symptoms, interpersonal sensitivity, anxiety, hostility, terror, paranoia, and psychosis (*P* < 0.05; Table 3).

**Table 3:** Partial correlation analysis between occupational stress and mental health.

**Variables**	**SCL-90** **total score**	**Somatization**	**Forced** **symptoms**	**Interpersonal** **sensitivity**	**Depression**	**Anxiety**	**Hostile**	**Phobia**	**Paranoid**	**Psychotic** **symptoms**
Occupational role	0.230^*^	0.231^*^	0.219^*^	0.207^*^	0.250^*^	0.226^*^	0.191^*^	0.207^*^	0.213^*^	0.173^*^
Personal strain	0.465^*^	0.451^*^	0.456^*^	0.433^*^	0.462^*^	0.445^*^	0.396^*^	0.409^*^	0.411^*^	0.388^*^
Personal resources	−0.051^*^	−0.053^*^	−0.038^*^	−0.079^*^	−0.017	−0.040^*^	−0.034^*^	−0.046^*^	−0.034^*^	−0.117^*^

* *There is a correlation between the two variables, p < 0.05*.

### Multivariate Analysis of Mental Health Related Factors

In order to analyze the factors affecting the psychological health of the oil workers in the desert, psychological abnormality was taken as the dichotomous dependent variable, while gender, ethnicity, age, length of service, type of work, educational level, professional title, shift work, monthly income, marital status, smoking, occupational task, individual stress responses, and individual coping resources were taken as independent variables. Multiple classification variables were set as dummy variables (Table 4), and multiple logistic regression analysis was performed. The results showed that the female gender, participants aged 45 years or above (relative to ≤30 years old), individual stress responses, occupational stress, external effort, internal investment, and high effort-low return were risk factors for mental health, while participants of Han nationality, oil transportation (relative to drilling), individual's ability to cope with strain, and work rewards were identified as protective factors for mental health (Table 5).

**Table 4:** Variable assignment.

**Variables**	**Name**	**Assignment**
Y	SCL-90 total score	Psychological abnormal = 0, Psychological normal = 1
X1	Gender	Male = 0, Female = 1
X2	Ethnicity	Minority = 0, Han = 1
X3	Age group, year	≤30 = 0, 30~45 = 1, >45 = 2
X4	Type of work	Drilling = 0, Extract oil = 1, Oil transportation = 2, Stoker hot note work = 3
X5	Working years	≤10 = 0, 10~20 = 1, >20 = 2
X6	Educational level	Associate's degree or below = 0, Bachelor's degree or higher = 1
X7	Professional title	Primary = 0, Secondary = 1, Senior = 2
X8	Shift	Fixed day shift = 0, Shift work = 1
X9	Monthly family income	≤3,500 = 0, >3,500 = 1
X10	Marital status	Single = 0, Married = 1, Widowed/divorced = 2
X11	Smoking	No = 0, Yes = 1
X12	Occupational stress	Low occupational stress = 0, Moderate occupational stress = 1,High occupational stress = 2
X12	ERI index	Low effort–high return = 0, High effort–low return = 1

**Table 5:** Logistic regression of the influencing variables of mental health.

**Variables**	**Group**	**β**	***S.E.***	***Wald***	***P***	***OR***	**95% CI**
Gender	Female	0.315	0.127	6.112	**0.013**	1.370	1.067 ~ 1.757
Ethnicity	Han	−0.409	0.11	13.718	**<0.001**	0.664	0.535 ~ 0.825
Age group, year	30 ~	0.006	0.216	0.001	0.978	1.006	0.659 ~ 1.536
	45 ~	0.351	0.123	8.116	**0.004**	1.420	1.116 ~ 1.808
Type of work	Extract oil	0.057	0.111	0.259	0.611	1.058	0.851 ~ 1.317
	Oil transportation	−0.729	0.164	19.796	**<0.001**	0.483	0.350 ~ 0.665
	Stoker hot note work	0.106	0.152	0.491	0.483	1.112	0.826 ~ 1.497
Working years	10 ~	0.142	0.163	0.756	0.385	1.153	0.837 ~ 1.588
	20 ~	0.093	0.161	0.334	0.564	1.098	0.800 ~ 1.505
Educational level	Bachelor's degree or higher	−0.032	0.131	0.061	0.806	0.968	0.748 ~ 1.253
Professional title	Secondary	−0.132	0.11	1.429	0.232	0.877	0.707 ~ 1.088
	Senior	0.081	0.128	0.404	0.525	1.085	0.844 ~ 1.395
Shift	Shift	0.134	0.104	1.647	0.199	1.143	0.932 ~ 1.402
Monthly family income	>3,500	0.069	0.106	0.415	0.519	1.071	0.869 ~ 1.319
Marital status	Married	−0.41	0.241	2.903	0.088	0.663	0.414 ~ 1.064
	Widowed/divorced	−0.276	0.161	2.942	0.086	0.759	0.554 ~ 1.04
Smoking	Yes	0.065	0.121	0.291	0.589	1.067	0.842 ~ 1.353
Occupational stress	Moderate occupational stress	−1.161	0.32	13.167	**<0.001**	0.313	0.167 ~ 0.586
	High occupational stress	−0.71	0.101	49.671	**<0.001**	0.492	0.404 ~ 0.599
Occupational role questionnaire	–	−0.001	0.003	0.181	0.671	0.999	0.994 ~ 1.004
Personal strain questionnaire	–	0.07	0.004	338.616	**<0.001**	1.073	1.065 ~ 1.081
Personal resources questionnaire	–	−0.017	0.002	50.694	**<0.001**	0.983	0.979 ~ 0.988

*The bold font means statistically significant*.

### Correlation Analysis Between the *DRD2* Gene and Mental Health

#### Comparison of Mental Health Scores Among Different DRD2 Genotypes

No statistically significant differences were found in the total mental health score and the scores of various factors among the *DRD2* gene rs1799732 genotypes (*P* > 0.05), and no differences were observed in the total mental health score, somatization, obsessive symptoms, interpersonal sensitivity, depression, anxiety, hostility, fear, paranoia, and psychosis scores corresponding to the *DRD2* gene rs1799732 genotypes. There were statistically significant differences in the scores of depression and paranoia among different rs1800497 genotypes (*P* < 0.05). According to the mean rank, the depression and paranoia scores of the AA genotype group were the highest, followed by the GA genotype group, while the scores of the GG genotype group were the lowest (Table 6).

**Table 6:** Comparison of mental health according to different *DRD2* genotypes.

	**rs1799732**			**rs1800497**		
	**DD**	**ID**	**II**	**AA**	**GA**	**GG**
SCL-90 total score	154.28 ± 59.77	157.08 ± 53.70	160.58 ± 58.88	162.36 ± 57.47	160.30 ± 57.08	152.85 ± 61.53
Somatization	20.56 ± 8.92	21.36 ± 8.22	22.30 ± 8.65	22.35 ± 8.51	22.12 ± 8.54	21.52 ± 8.88
Forced symptoms	16.56 ± 7.63	16.91 ± 6.31	17.27 ± 7.21	17.56 ± 7.20	17.21 ± 6.86	16.33 ± 7.31
Interpersonal sensitivity	15.11 ± 6.94	16.04 ± 5.67	16.44 ± 6.29	16.60 ± 6.08	16.48 ± 6.13	15.35 ± 6.52
Depression	24.56 ± 9.48	24.28 ± 8.25	24.95 ± 8.77	25.24 ± 8.71^*^	24.98 ± 8.56	23.35 ± 8.92
Anxiety	16.56 ± 6.64	17.69 ± 6.13	17.94 ± 6.82	18.13 ± 6.76	18.01 ± 6.54	16.88 ± 6.95
Hostile	9.33 ± 4.33	9.75 ± 3.64	10.01 ± 4.25	10.04 ± 4.08	10.07 ± 4.15	9.39 ± 4.24
Phobia	10.67 ± 4.77	11.95 ± 4.89	12.18 ± 5.02	12.16 ± 4.86	12.27 ± 4.92	11.53 ± 5.49
Paranoid	10.33 ± 4.18	10.69 ± 3.83	11.06 ± 4.06	11.16 ± 3.80^*^	11.02 ± 3.98	10.49 ± 4.57
Psychotic symptoms	16.67 ± 5.63	17.04 ± 5.97	17.69 ± 6.75	17.85 ± 6.64	17.52 ± 6.45	17.03 ± 7.03

*Comparison of mental health scores corresponding to different DRD2 loci genotypes*,

* *P < 0.05*.

#### Distribution of DRD2 Genotypes and Alleles for Different Mental Health Conditions and Their Effects on Mental Health

There were two alleles at rs1799732 of the *DRD2* gene, which were wild allele I and mutant allele D. The genotypes included homozygous wild-type (II), heterozygous type (ID), and homozygous mutant (DD). The results of the statistical analysis showed no significant difference in the distribution of genotypes and alleles at the rs1799732 locus between the group with psychological abnormalities and the group with no psychological abnormalities (*P* > 0.05). The multivariate logistic regression analysis showed that, compared with the II genotype, the ID genotype, DD genotype, and the mutant (DD+ID) had no effect on mental health (*P* > 0.05).

There were two alleles at rs1800497 of the *DRD2* gene, which were wild type allele G and mutant allele A. There were three genotypes: GG (homozygous wild type), GA (heterozygous type), and AA (homozygous mutant). The results of the statistical analysis showed that the distribution of genotypes and alleles at the rs1800497 locus was significantly different between the psychologically abnormal group and the psychologically normal group (*P* < 0.05). Multiple logistic regression analysis showed that, compared with the GG genotype, the AA genotype (OR = 1.907, 95% CI: 1.186–3.066) was identified as the genotype that was specific to susceptibility for mental health problems, and the A allele (OR = 1.293, 95% CI: 1.042–1.605) was associated with an increased risk of developing mental health problems (Table 7).

**Table 7:** The distribution of the *DRD2* genotype and allele, and their effects on mental health.

**Gene loci**	**Genotype**	**Psychologically** **abnormal**	**Psychologically** **normal**	***χ*** ^2^	***P***	**OR 95 (%CI)**
rs1799732	II	258	302	0.711	0.701	1.000
	ID	56	71			1.709 (0.423 ~ 6.900)
	DD	3	6			1.083 (0.735 ~ 1.596)
	DD + ID	59	77	0.319	0.631	1.079 (0.787 ~ 1.479)
	I	572	675	0.507	0.483	1.000
	D	62	83			1.134 (0. 802 ~ 1.606)
rs1800497	GG	117	121	**7.560**	0.022^*^	1.000
	GA	164	187			1.103 (0.793 ~ 1.533)
	AA	36	71			**1.907 (1.186 ~ 3.066)**
	AA + GA	200	258	1.904	0.173	1.247 (0.911 ~ 1.708)
	G	398	429	**5.467**	0.021^*^	1.000
	A	236	329			1.293 (1.042 ~ 1.605)

* *Comparison of the genotype and allele frequency of GG, GA, and AA between the psychologically abnormal group and psychologically normal group (P < 0.05)*.

*The bold font means statistically significant*.

## Discussion

The study aimed to explore the relationship between occupational stress, the *DRD2* gene, and the mental health status of oil field workers in the arid desert environment of Xinjiang. The results showed that there were statistically significant differences in the total mental health scores and the scores of various factors among different stress intensity groups. Higher levels of occupational stress were associated with poorer mental health level. Relevant studies have also shown that, in groups such as firefighters (35), police officers (36), medical staff (37), and workers (38), the level of occupational stress affected the mental health status to different degrees. In addition, other studies (39, 40) have shown that during the extraordinary period of the COVID-19 pandemic, anesthesiologists, community workers, and other groups also experienced a high level of occupational stress, which led to anxiety, depression, and the onset of other symptoms. This may be related to the special working environment of oil workers who are subjected to a harsh and unaccommodating climate, which is only hospitable to a relatively boring and monotonous life. Therefore, it is imperative to develop targeted intervention measures to alleviate occupational stress, promote mental health, and further explore the main risk factors affecting occupational stress in the key population who experience a high level of occupational stress.

In respect to the partial correlation analysis which examined the relationship between occupational stress and mental health status, after controlling for confecting factors (e.g., gender, ethnicity, age, length of service, type of work, educational level, professional title, shift status, monthly income, marital status, and smoking status), occupational tasks and individual stress responses were positively correlated with total mental health scores, somatization, obsessive symptoms, interpersonal sensitivity, depression, anxiety, hostility, terror, paranoia, and psychosis. Individual coping resources were negatively correlated with total mental health scores, somatization, obsessive-compulsive symptoms, interpersonal sensitivity, anxiety, hostility, fear, paranoia, and psychosis. This finding is similar to the results of the study conducted by Moreno Fortes et al. (41) which found that occupational stress was negatively correlated with positive mental health and lower psychopathological symptoms, while job burnout played a mediating role between occupational stress and mental health. Therefore, arranging work tasks in a reasonable manner can increase an individual's coping resources, and intervention measures can be developed to alleviate occupational stress among oil workers in order to improve their psychological health.

Relevant studies have also shown (42, 43) that occupational stress and effort-reward imbalance are risk factors for the occurrence of psychological disorders in workers, which is similar to the results of this study. This study found that individual stress reactions, occupational stress, external effort, internal investment, and high effort-low return were the main risk factors for the mental health of oil workers. In addition, compared with men, the mental health status of women was poorer. This may be related to the special working environment and the nature of the work, which tends to result in greater work pressure for female employees. In this regard, some studies have shown that health education programs, based on a work-related stress model, can alleviate work-related pressure among female employees (44). Individual resilience and job rewards were identified as protective factors of mental health. Flexible working arrangements and a better working environment can relieve the working pressure of employees (45). However, in the arid desert environment of Xinjiang, the working environment of oil field workers is relatively challenging. Work tasks can be arranged to allow for greater flexibility, and efforts can be made to improve the working environment. Nonetheless, further research is necessary to determine whether such measures can relieve occupational stress among oil workers, and to identify other ways of enhancing their mental health.

Previous studies have shown that the *DRD2* gene has a certain correlation with mental health (46–48). This study also found that the distribution of the *DRD2* gene genotypes and alleles at rs1800497 was significantly different between the group with psychological abnormalities and the group with no psychological abnormalities (*P* < 0.05), which indicated that the various genotypes of the *DRD2* gene at rs1800497 may be related to the mental health of oil workers. This result is similar to the research results of some scholars (49, 50), who proposed that the *DRD2* gene is associated with depression and other mental health problems. However, other studies have shown (51) that the genotype of the *DRD2* gene at rs1800497 is not associated with the remission of schizophrenia symptoms, which may be due to different sample sizes and regional differences. Multiple logistic regression analysis showed that, compared with the GG genotype, the AA genotype (OR = 1.907, 95% CI: 1.186–3.066) was specific to a susceptibility for mental health problems, and A allele (OR = 1.293, 95% CI: 1.042–1.605) was associated with an increased risk of developing mental health problems. There were statistically significant differences in the scores of depression and paranoia among different genotypes of rs1800497 (*P* < 0.05). This finding is similar to those of Avinun et al. (52), Galyamina et al. (53), suggesting that different genotypes of the *DRD2* gene at rs1800497 are associated with depression. According to the mean rank, the depression and paranoia scores of the AA genotype group were the highest, followed by the GA genotype group, while the GG genotype group were the lowest. These findings suggest that the AA genotype may affect mental health problems to a certain extent, and occupational stress factors may influence the onset of mental illness and the progression of the disorder.

In recent years, many experts in psychology, brain neuroscience and other fields have begun to explore how genes affect the neural structure and function of the brain, and result in mental illness (54). Studies have shown a highly polygenic pattern of inheritance, which may be another important factor in the heterogeneity of depression (55). Therefore, understanding how genetic variations affect brain circuits, and thus individual psychology, requires more in-depth and integrated research. In future studies, the effects of occupational stress, adverse genetic and environmental interactions and other risk factors on the mental health of oil workers need to be further explored. The effects of various risk factors, such as adverse gene and environment interaction, on the mental health of oil workers need to be further examined.

This study demonstrated that occupational stress and the *DRD2* gene influenced the mental health level of oil field workers in the arid desert environment of Xinjiang. The effort-reward imbalance and occupational stress were identified as risk factors for mental health, while work rewards were a protective factor. High levels of occupational stress can lead to psychological disorders, such as depression, and adversely affect mental health. Therefore, measures should be taken to alleviate the occupational stress of oil workers and improve their job rewards, so as to improve their mental health. This study also found that, the AA genotype of the *DRD2* rs1800497 polymorphism is a genotype that is specific to susceptibility to mental health problems, and the A allele increased the risk of mental health problems.

## Conclusion

### Strengths and Limitations

This study investigated the relationship between occupational stress and mental health status, and revealed the relationship between psychological disorders and genetics, which few studies have examined. Even though this research was a cross-sectional study, a randomized sampling method was employed. Valid and reliable questionnaires (validated in Chinese) were used, which minimized bias. However, this study also had some limitations. First, due to the cross-sectional design, the results may have some shortcomings. Second, this study used a small sample size, and the participants were recruited from an oil field company in Xinjiang, which may not be representative of the entire population of oil workers in China. Finally, in this study, a self-rating scale was used to measure psychological disorders and occupational stress, which may lead to recall or report bias. In addition, we acknowledge that it is difficult to confirm the relationship between *DRD2* gene and psychological disorders solely on the basis of the results of this study. Further studies are needed to investigate whether the interaction between *DRD2* genotype and occupational stress contributes to an increased risk of psychological disorders.

### Directions of Further Research

Future studies could aim to conduct a cohort study to explore psychological problems caused by occupational stress among oil field workers in the arid desert environment of Xinjiang. Such studies can provide a more powerful theoretical basis for an in-depth study of occupational stress and the mental health of oil workers.

## Data Availability Statement

The original contributions presented in the study are included in the article/supplementary files, further inquiries can be directed to the corresponding author/s.

## Ethics Statement

The studies involving human participants were reviewed and approved by Xinjiang Medical University. The patients/participants provided their written informed consent to participate in this study.

## Author Contributions

TJ, GW, XL, and JL designed the study. TJ, GW, XL, and XY contributed to the acquisition, analysis, interpretation of data, involved in drafting the manuscript, and revising it for important intellectual content. All authors contributed substantially to the work presented in this paper, reviewed, and approved the final manuscript.

## Funding

This study was funded by the Nature Science Foundation of Xinjiang autonomous region (Grant No. 2018D01C146).

## Conflict of Interest

The authors declare that the research was conducted in the absence of any commercial or financial relationships that could be construed as a potential conflict of interest.

## Publisher's Note

All claims expressed in this article are solely those of the authors and do not necessarily represent those of their affiliated organizations, or those of the publisher, the editors and the reviewers. Any product that may be evaluated in this article, or claim that may be made by its manufacturer, is not guaranteed or endorsed by the publisher.
